# DSM-5 suicidal behavior disorder: a systematic review of research on clinical utility, diagnostic boundaries, measures, pathophysiology and interventions

**DOI:** 10.3389/fpsyt.2024.1278230

**Published:** 2024-01-23

**Authors:** Etinosa Oliogu, Anthony C. Ruocco

**Affiliations:** ^1^ Department of Psychological Clinical Science, University of Toronto, Toronto, ON, Canada; ^2^ Department of Psychology, University of Toronto Scarborough, Toronto, ON, Canada

**Keywords:** suicide, suicidal behavior disorder, DSM-5, self-harm, self-harm behavior

## Abstract

**Background:**

It has been a decade since Suicidal Behavior Disorder (SBD) was introduced in Section III of the DSM-5 under “Conditions for Further Study”. SBD is chiefly characterized by a self-initiated sequence of behaviors believed at the time of initiation to cause one’s own death and occurring in the last 24 months.

**Aims:**

To systematically review empirical studies on SBD to identify primary research themes and promising future research directions.

**Method:**

A search of empirical articles on SBD published between May 2013 and March 2023 was conducted according to the Preferred Reporting Items for Systematic Reviews and Meta-Analyses guidelines.

**Results:**

Screening of 73 records by two independent raters yielded 14 eligible articles. The primary research themes identified from these articles included clinical utility of SBD to predict future suicide risk, association of SBD with closely related disorders, psychometric properties of SBD measures, pathophysiology of SBD, and the effectiveness of interventions for people with SBD.

**Conclusion:**

Understanding of SBD has slowly progressed since its introduction a decade ago and has mainly been applied in research to define study groups displaying suicidal behavior. The clinical utility of SBD for predicting future suicide risk is low and more research is needed to understand measurement of the diagnosis and its distinctiveness from related disorders and other self-harming behaviors.

## Introduction

Suicide is a major public health concern (1). Over 700,000 people die due to suicide each year, not accounting for the number of suicide attempts that likely occur but go unreported (2). Over the years, research in the field of suicidology has identified several risk factors for suicidal behavior, including previous suicide attempts, psychiatric disorders, hopelessness, impulsivity, aggression, and childhood trauma (3). Despite this, predicting future suicidal behavior and treatment following a suicide attempt continues to be a significant challenge for individuals, families, mental health professionals, and researchers (3) due to the complex and ever-changing mechanisms of underlying suicidal behavior.

An ongoing conversation among researchers and mental health practitioners is whether suicidal behavior should be formulated as a distinct diagnosis in official psychiatric diagnostic nosologies. It is often discussed as a symptom in the context of another psychiatric disorder, such as major depressive disorder (MDD) or borderline personality disorder (BPD; 4). However, growing evidence highlights the potential for the introduction of a specific disorder of suicidal behavior because of the unique pathophysiology associated with suicide attempt and the clinical utility of such a diagnosis for predicting future suicide attempt (1, 4, 5). The American Psychiatric Association (6) proposed Suicidal Behavior Disorder (SBD), an attempt in the Diagnostic and Statistical Manual of Mental Disorders (DSM) to capture suicidality as a diagnosis rather than a clinical feature requiring attention.

The proposed criteria were put forth in Section III of the Fifth Edition of the DSM (DSM-5), under “Conditions for Further Study” (6, p.783). Diagnoses in this section are not yet intended for clinical use but instead are presented to encourage future research with common language and parameters (6). The hope is that following the accumulation of research supporting the incremental validity and clinical utility of these proposed conditions, they can be placed with the other official and clinically recognized mental disorders in Section II of the DSM in future editions.

According to the APA (6), SBD is characterized by a suicide attempt within the last 24 months (Criterion A). A suicide attempt is defined as “a self-initiated sequence of behaviors by an individual who, at the time of initiation, expected that the set of actions would lead to his or her own death” (6, p. 801). The act cannot meet the criteria for non-suicidal self-injury, that is, self-injury with the intention to relieve negative feelings or cognitive state in order to achieve a positive mood state (Criterion B) and cannot be applied to suicidal ideation or preparatory acts (Criterion C). If the attempt occurred during a state of delirium or confusion or solely for political or religious objectives, then SBD is ruled out (Criteria D & E). SBD, current, is given when the suicide attempt occurred within the last 12 months, and SBD, in early remission, when it has been 12-24 months since the last attempt.

March 2022 marked the APA’s latest release, the DSM-5-Text Revision (DSM-5-TR), wherein SBD did not develop as expected or hoped for (nor any separate diagnosis for suicidal behavior) by some researchers and practitioners (1, 7). Indeed, arguments to include SBD in the DSM-5 centered on evidence to support its reliability and validity, and the potential to improve approaches to identification of suicidal behavior through greater integration with clinical practice (4, 8). The identification of suicidal behavior in mental health care service settings is not without its potential drawbacks, however, especially given the stigma attached to the behavior (9, 10). Furthermore, criticisms of the SBD diagnosis, specifically, include the clinical utility and specificity of the 24-month timeframe during which a suicide attempt has occurred (Criterion A), and the boundary between non-suicidal self-injury and suicide attempt (Criterion B; 11).

Ultimately, SBD was removed as a condition for further study in Section III and instead placed under “Other Conditions That May Be a Focus of Clinical Attention” in Section II. The conditions in this section are meant to draw clinician attention to the presence and breadth of additional issues routinely encountered in clinical practice and provide a procedure for their systematic documentation (12). Diagnostic codes are provided for current suicidal behavior, initial and subsequent encounters, and lifetime history. Specifiers for non-suicidal self-injury, current or history, are also provided. These changes appear to be in line with the International Classification of Diseases—11^th^ Revision, which includes “Aspects of intentional self-harm events”, for example, under so-called “Extension Codes” and within the “Dimensions of External Cause” classification scheme.

According to the APA (13), the rationale for the exclusion of SBD from the DSM-5-TR was based on concerns that the proposed disorder did not meet the criteria for a mental disorder but instead constituted a behavior with diverse causes. The proposed diagnosis was also criticized for having limited clinical utility because it did not provide information on the current risk for suicide; it only described recent suicide history. Another influence on the exclusion of SBD was the view that a diagnostic label based on a single past event could lead to increased stigma and discrimination towards people with a history of suicidal behavior (13). The DSM steering committee also believed that the deletion of SBD would not hinder further research activity related to suicidality. The APA (12) suggested that a diagnostic code for the presence of suicidal behavior would help improve documentation of these behaviors when occurring with other disorders and mitigate the risk of future suicide attempts or death. This is in addition to encouraging research targeting the treatment of suicidal behavior specifically rather than as a symptom of an associated condition. Whereas questions about the validity and clinical utility of the diagnosis primarily concerned future suicide risk and the potential for stigma and discrimination, a comprehensive review of empirical studies addressing these and other characteristics of SBD is not yet available.

This systematic review aims to summarize the research on SBD conducted over the last decade, since the publication of the DSM-5 in 2013. It could be argued that many studies of recent suicidal behavior might qualify for such a review; however, the intention of the present systematic review is to draw specific attention to SBD as conceptualized in the DSM-5, in part to develop an understanding of the extent to which the introduction of the diagnosis has stimulated research in the last 10 years. Furthermore, as the diagnosis was removed from the DSM-5-TR, a contemporary synthesis of empirical studies that identifies primary research themes and outlines the scope of such work may be informative and highlight potential research areas warranting possible further scrutiny (e.g., to modify or refine the diagnosis to improve its clinical utility and validity).

## Method

### Inclusion and exclusion criteria

Studies were eligible for inclusion in the narrative review if they were original empirical reports and reported on the SBD diagnosis as a major focus of the study. To capture as many relevant studies as possible, the range of eligible topics and outcomes was intentionally broad. Given the APA’s description of the rationale for excluding SBD from DSM-5-TR, research was nevertheless expected to be identified in the areas of clinical utility (e.g., predicting future suicidal behavior), stigma and discrimination, pathophysiology, and psychometric properties of SBD measures.

### Search strategy

A literature search for empirical articles was conducted on March 13, 2023, through PubMed and PsycINFO. A combination of the following key terms was used: “Suicidal behavior disorder”, “suicidal behavior disorder”, and “DSM.” The search included studies published between May 2013 and March 2023.

### Study selection

**Figure 1:**
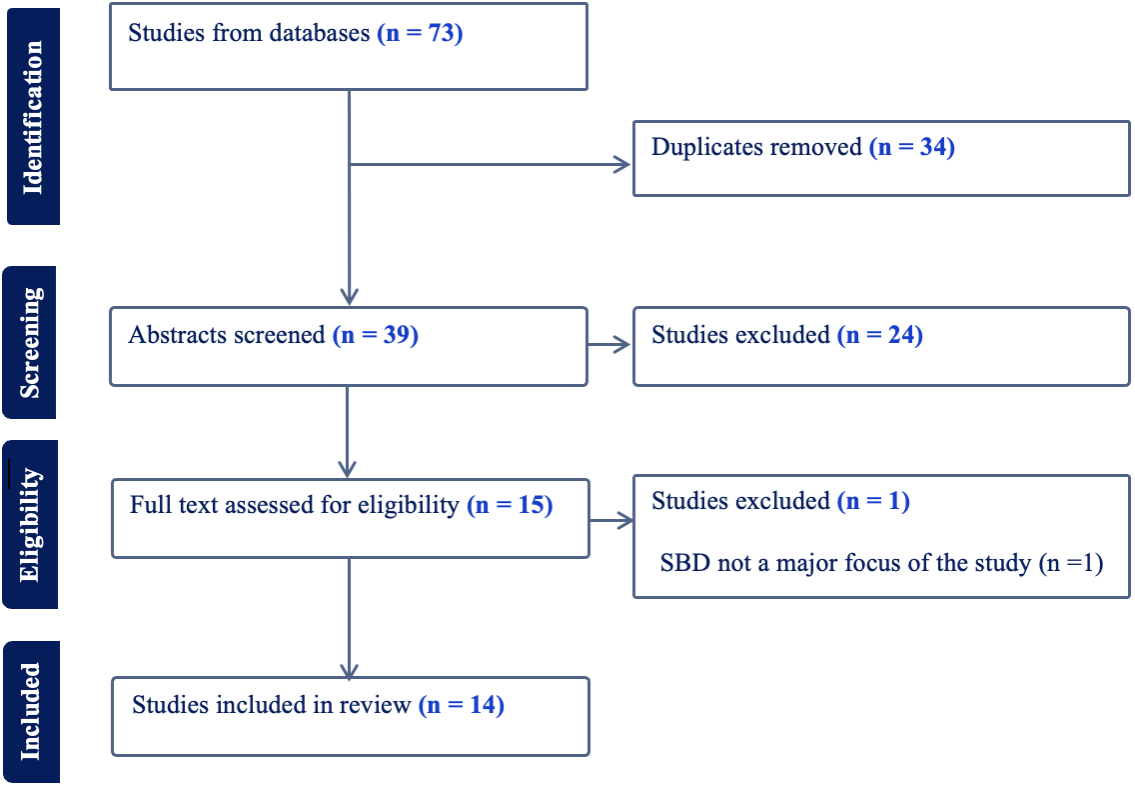
Screening of articles according to PRISMA guidelines.

Two authors (EO, AR) independently screened selected materials using the Covidence systematic review management software. Articles were screened and reported according to the methods outlined by the Preferred Reporting Items for Systematic Reviews and Meta-Analyses (PRISMA). Articles were first assessed by title and abstract according to the eligibility criteria, and promising articles then underwent full-text review. Screening results discrepancies between authors were discussed until a consensus was reached (**Figure 1**).

### Data collection and analysis

The study aim, population characteristics, and key findings were recorded for each article and grouped by primary theme. As anticipated, given the diversity of the aims and methods of the studies identified, they were not suitable for a quantitative synthesis (e.g., meta-analysis). Accordingly, a qualitative review of the findings was carried out and organized according to the common themes that emerged from the studies.

## Results

The search returned 73 records (13 duplicates were subsequently removed), of which 14 were deemed eligible for inclusion in the systematic review, including one article that was discussed further due to a discrepancy between the rating authors and was excluded. Articles judged to be irrelevant for the systematic review through the screening and rating process included review articles/commentaries and empirical reports for which SBD was not stated as the primary focus of the study. Scrutiny of the topics of the articles suggested that they fell within five general areas: (a) clinical utility; (b) validity based on boundaries with related conditions; (c) psychometric properties of SBD measures; (d) pathophysiology of SBD; and (e) interventions for people with SBD. Accordingly, the reporting of the results of these studies is organized around these topics.

### Clinical utility

Clinical utility is defined as the extent to which a diagnosis assists clinical decision-making by fulfilling the various clinical functions of a psychiatric classification system (14). Two studies investigated SBD as a predictor of future suicide risk, addressing one aspect of clinical utility, communicating clinical information to practitioners (14). Lasisi and colleagues (15), in their study on the prevalence and correlates of suicide risk in incarcerated youth, found that of 262 incarcerated youth in northern Nigeria, SBD had a prevalence rate of 7.6% and was not significantly associated with suicide risk. The absence of current depression, previous incarceration, increasing age, and family circumstances were more predictive of suicide risk.

In an earlier study, Lübbert and colleagues (3) found that their sample of 212 people with current SBD represented a very heterogeneous group, with those at risk of future suicidal behavior demonstrating severity on key clinical features not captured by the SBD diagnosis (i.e., psychopathology, suicidal ideation, hopelessness, genetic and environmental risk factors, and specific personality trait). These studies, in addition to relatively few studies investigating the clinical utility of SBD, raise the question of what, if any, additional information is provided to practitioners by an SBD diagnosis. The authors of both articles named the cross-sectional design as a limitation of their studies, limiting the temporal conclusions that can be drawn.

### Diagnostic boundaries

NSSID is another proposed diagnosis under “Conditions for Further Study” in DSM-5 and, unlike SBD, was retained in the DSM-5-TR. NSSID is characterized by intentional self-inflicted damage to the body’s surface that is likely to induce bleeding, bruising or pain occurring on five or more days over the last year. The intention of these behaviors is not to die but to induce relief, achieve a positive state, resolve interpersonal difficulty, or a combination of these (7).

SBD has been studied in relation to NSSID and BPD because of the prevalence of suicidal behavior in both disorders. Groschwitz and colleagues (16) investigated the association between NSSID and SBD in 111 adolescent psychiatric inpatients and found that the two tend to co-occur, with NSSID acting as a strong risk factor for the occurrence of SBD. Similar results were found by Szewczuk-Bogusławska and colleagues (17). There was high co-occurrence of SBD and NSSID in their sample of 196 adolescent girls with conduct disorder, and a diagnosis of NSSID, with a minimum of eight days of self-injury engagement within the last 12 months, significantly predicted the risk of SBD.

SBD has also been studied with BPD. Ducasse and colleagues (5) compared the psychological and clinical traits of suicidal vulnerability in 92 SBD patients, both with and without BPD. They found that scores in clinical and psychological traits of suicidal vulnerability increased along a dimension from healthy controls to SBD patients occupying the intermediate position, and comorbid SBD and BPD were associated with particularly high scores of suicidal vulnerabilities. The authors suggest that BPD could act as a specifier for SBD diagnoses.

Consistent with this, Levine et al. (18), in a similar investigation on BPD and NSSID, also found that although the number of serious suicide attempts was primarily associated with BPD, there were some incidents of suicide attempts reported by individuals who met no diagnostic criteria for BPD. This again suggests the presence of an intermediate group with clinically relevant suicidal attempts, as described by SBD, but who do not otherwise meet the criteria for BPD. SBD captures this group that may be missed or may warrant targeted treatment.

### Psychometric properties of SBD measures

The Self-Injurious Thoughts and Behaviors Interview (SITBI) is a structured clinical interview that assesses the presence, frequency, and characteristics of a wide range of self-injurious thoughts and behaviors (19). Fischer and colleagues (20) assessed the psychometric quality and properties of the German translation of the SITBI (SITBI-G) in 111 adolescent inpatients. They employed Cohens Kappa to evaluate test-retest and interrater reliability of the SITBI-G for assessing SBD. They found that the SITBI-G showed moderate to good test-retest reliability when assessing for SBD (k =.64) and current SBD (k =.52). Additionally, it exhibited excellent interrater reliability for SBD (k=1.00) and current SBD (k=1.00).

Lee et al. (21) found similar results when developing and providing initial psychometric validation for the Korean version (SITBI-K) to assess SBD in 108 undergraduate subclinical research participants. Both author groups concluded that a diagnosis of SBD can be established using their language version of the SITBI. The limitation of both studies is the lack of information on the translation procedures utilized to ensure comparability to the English version. Additionally, the long-form version of the SITBI does not directly assess for functional impairment, a criterion for an SBD diagnosis. This was pointed out by Fischer et al. (20) but not Lee et al., which would limit both studies’ results and interpretation.

### Pathophysiology

The pathophysiology of suicidal behavior is complex and involves interactions among multiple biological systems (for a review, see 22). Research on the pathophysiology of SBD, more specifically, has until now exclusively centered on endocrinological markers, and these findings appear to be reported by the same or overlapping research groups. In the first of three articles on related topics, Duval et al. (23) investigated thyrotropin (thyroid-stimulating hormone; TSH) and prolactin (PRL) responses to protirelin (thyrotropin-releasing hormone; TRH) stimulation tests in depressed inpatients with either current SBD (last suicide attempt within the last one year) or SBD in early remission (last suicide attempt in the last 1-2 years), as well as non-psychiatric controls. Participants with SBD in early remission did not differ from controls across TSH and PRL measurements. However, compared to controls and SBD participants in early remission, the current SBD group showed lower changes (following TRH injection) in TSH at the first measurement time (2300 h) and lower differences in changes in TSH response between the two measurement times (2300 h and 0800 h). Among participants with current SBD, the latter values were also significantly negatively correlated with lethality ratings of the most recent suicide attempt. Additionally, free thyroxine levels were lower in current SBD compared to controls. Some of the findings were accentuated in a subgroup of participants with current SBD who were classified as violent suicide attempters. The findings were interpreted to support the theory that individuals with current SBD show an inadequate homeostatic mechanism implicating the TRH response to lowered serotonin activity.

In a separate report, Duval et al. (24) investigated multiple hormonal responses to apomorphine (APO), which is a dopamine receptor agonist, and protirelin (TRH), in depressed inpatients with current or in early remission SBD, and non-psychiatric controls. Similar to the pattern of findings in Duval et al. (23), participants with SBD in early remission did not differ from controls in their responses to APO and TRH tests, although there were various indicators of adrenocorticotropic hormone and APO-induced growth hormone dysregulation. Duval et al. (25) grouped participants in the same manner and studied their prolactin responses to APO and protirelin at different time points. Baseline prolactin measurements did not differ across the three groups, and comparable to the results of prior studies, SBD participants in early remission showed no differences from controls across the various measurements. However, participants with current SBD displayed lower prolactin suppression values than controls, and smaller differences in prolactin change values between the two testing times (2300 h and 0800 h); the co-occurrence of these observations was higher in patients whose most recent suicide attempt was violent and highly lethal. Taken together, the results suggest a dysregulation of the hypothalamic-prolactin axis in depressed patients with current SBD.

### Interventions

The efficacy of established interventions to treat SBD specifically has been investigated and seen promising results. Ducasse and colleagues (26) conducted a pilot study investigating the usefulness of an add-on Acceptance and Commitment Therapy (ACT) group program to decrease suicidal ideation in 35 patients with current SBD. They found that an adjunctive ACT group program decreased suicidal ideation through increasing acceptance skills and meaning of existence and reducing the impact of modifiable suicidal risk factors (i.e., hopelessness, psychological pain, quality of life) in patients with current SBD. In a randomized controlled trial (RCT) conducted a few years later in 40 adults with current SBD, the authors found that the rate of change in ACT for suicidal ideation was higher than in the relaxation group (27). Both author groups concluded that ACT might be an effective intervention for patients with SBD. Another aspect of clinical utility discussed by First and colleagues (14) is improving clinical outcomes; a diagnosis should assist in choosing effective interventions that achieve this. The focus on suicidal ideation in these studies limits its interpretation and effectiveness for people with SBD because suicidal ideation is not a defining feature in the diagnosis and represents somewhat of an exclusion criterion.

Henrion and colleagues (28) took a different approach by investigating the effectiveness of a psychoeducational program for managing patients with current SBD. They found that when compared to a relaxation group, although both groups benefited from their respective groups, the psychoeducation program had more profound implications for daily functioning through specific processes of targeting suicidal risk (e.g., developing an internal locus of control and acquiring scientific knowledge on suicidal behavior) and reducing stigma, the psychoeducation program may represent a promising intervention for suicide prevention. The generalizability of these results is limited due to a small sample size (n = 18).

## Discussion

The present systematic review comprehensively summarized original empirical studies of SBD as defined in the DSM-5, Section III. While the number of studies identified in the review was relatively low, the scope of the work represented a range of primary themes, including clinical utility (e.g., suicide risk), diagnostic validity compared to related diagnoses, psychometric characteristics of SBD measures, pathophysiology, and psychological interventions. The majority of research on SBD located by the search was conducted outside of North America. Only one study (18) was conducted in the United States. The remaining studies on SBD were conducted in several countries, including Nigeria, Germany, Switzerland, Poland, Korea, and France. The research was conducted with similar frequency in adolescents and adults. All but two studies were conducted with inpatients, presumably representing more extreme cases of SBD requiring hospitalization.

Consistent with the rationale for change provided by the DSM-5-TR steering committee, the clinical utility of SBD may be judged as low, as the features most predictive of suicide risk, such as cognition and psychopathology (29, 30), are not fully captured in SBD. Additionally, many studies used the diagnosis to demarcate a timeframe (i.e., less than one year since the last suicide attempt) rather than clinical characteristics related to the suicide attempt that may be informative for intervention and possibly safety planning (e.g., medical lethality of the last suicide attempt). Relatedly, emerging research indicates that current (past year) SBD among depressed inpatients (especially those with a recent violent and high-lethality attempt) may be linked to a specific profile of endocrinological markers compared to those with SBD in early remission. These findings suggest that the timing of the most recent suicide attempt and associated clinical characteristics may differentiate subtypes of SBD, at least at a pathophysiological level. Second, NSSID was found to be a strong predictor of SBD and will likely continue to be studied in relation to suicidal behavior in the absence of SBD. Although SBD is suggested to provide a diagnosis for subthreshold clinically significant presentation, as seen in work related to BPD, more research is needed to support this and a potential reformulation of the diagnosis to suit this specific function. It is also possible that the new diagnostic codes for suicidal behavior and non-suicidal self-injury may capture this intermediate group.

At first glance, the psychometric properties of the translated versions of the SITBI for assessing SBD seem promising, but when considering that full SBD criteria are not covered in the measure, their results should be cautiously interpreted and generalized. Finally, ACT and a psychoeducational program were administered in a group of individuals with SBD in RCTs, and both were shown to be more effective than relaxation controls. Conclusions that can be drawn from the existing literature on SBD in several respects are limited and likely contributed to its removal from the DSM-5-TR.

### Future directions

While there is no clear future direction for SBD as a diagnosis, many researchers in the field argue for the adoption of a diagnostic entity for suicidal behavior for reasons related to clinical utility and the implications of conceptualizing suicidality as a symptom rather than a disorder (1, 4). The present review identified a relatively small number of studies on SBD, possibly because the diagnostic formulation of SBD in the DSM-5 did not stimulate sufficient interest in studying the disorder (11, 31). Nevertheless, the emerging research on SBD highlights topics that may be worthy of future study. For example, SBD could serve as an anchoring diagnosis for studies aimed at reducing suicide risk and related symptoms and functional impairment.

## Author contributions

EO: Conceptualization, Investigation, Methodology, Project administration, Visualization, Writing – original draft. AR: Methodology, Supervision, Validation, Writing – review & editing.
